# *Salmonella* mediated the hemagglutinating virus of Japan-envelope transfer suppresses tumor growth

**DOI:** 10.18632/oncotarget.17037

**Published:** 2017-04-11

**Authors:** Che-Hsin Lee, Tomoyuki Nishikawa, Yasufumi Kaneda

**Affiliations:** ^1^ Department of Biological Sciences, National Sun Yat-sen University, Kaohsiung, Taiwan; ^2^ Department of Medical Research, China Medical University Hospital, China Medical University, Taichung, Taiwan; ^3^ Division of Gene Therapy Science, Graduate School of Medicine, Osaka University, Osaka, Japan

**Keywords:** Salmonella, targeted therapy, polymer, hemagglutinating virus of Japan-envelope

## Abstract

*Salmonella* can target to tumor microenvironments after systemic treatment. The hemagglutinating virus of Japan-envelope (HVJ-E) induced apoptosis in tumor cells without toxicity in normal cells. Current HVJ-E therapeutic strategies, aimed at using HVJ-E for intratumor treatment, have shown great promise in animal models but have achieved only limited systemic treatment. The purpose of this study was to investigate the modulation of the anti-tumor efficiency of HVJ-E by coating the particles with poly (allylamine hydrochloride) (PAH), designated as P-HVJ-E. Treatment with P-HVJ-E resulted in decreased hemagglutinating activity and maintained tumor cell-selective apoptosis and anti-tumor immunity. The use of *Salmonella* as a coating for P-HVJ-E (PHS) enhanced the antitumor activity and maintained the tumor-targeting activity. Treatment with PHS resulted in delayed tumor growth in tumor-bearing mice. Furthermore, a Western blot assay of the tumors revealed that HVJ-E targeted to the tumor after systemic treatment with PHS. These results indicate that *Salmonella* coating viral particles may provide a new approach for tumor therapy.

## INTRODUCTION

Currently, tumor treatment suffers from a lack of specific tumor targeting agents [1]. The non-pathogenic facultative anaerobic bacteria, *Salmonella*, have been found to specifically target to tumor sites [2–6]. *Salmonella* are able to colonize small metastatic and larger tumors, because they can grow under aerobic and anaerobic conditions [7–10]. *Salmonella* are already widely used in a broad range of human and mouse tumors [11–19]. Over the decades, variants of tumor proteins and pathways inhibited by *Salmonella* have been intensively studied [20], such as indoleamine 2, 3-dioxygenase 1 (IDO-1), leading to immune tolerance [21]. Based on the observation of hypoxic regions in the tumor, *Salmonella* could specifically target tumor tissue [22]. *Salmonella* have been demonstrated as potential tumor-targeting vectors for therapeutic agent delivery [23]. The advancement of cancer research may rely on a rapid shift in the engineering of oncolytic viral systems because this method has shown high efficiency and less side effects for tumor therapy, especially the hemagglutinating virus of Japan-envelope (HVJ-E) [24, 25].

Previously, the masking of *Salmonella* with a polymer reduced the antigenicity of *Salmonella* [26]. Herein, we attempted to encapsulate the hemagglutinating virus of Japan-envelope (HVJ-E) into poly (allylamine hydrochloride) (PAH) and change the surface charged of HVJ-E [26]. Polymers shield HVJ-E from the hemagglutination activity after systemic administration [26]. The presence of lipopolysaccharide (LPS) in the cell wall of gram-negative bacteria results in the negatives surface charged of *Salmonella*. The negatively charged bacterial surfaces can absorb the positively charged polyelectrolytes to form a complex [26]. Here, this study demonstrates that the oncolytic virus delivery into the tumor site by using tumor-targeting *Salmonella* to enhance the therapeutic index.

## RESULTS

### The encapsulation of HVJ-E into polyelectrolyte shells

**Figure 1:**
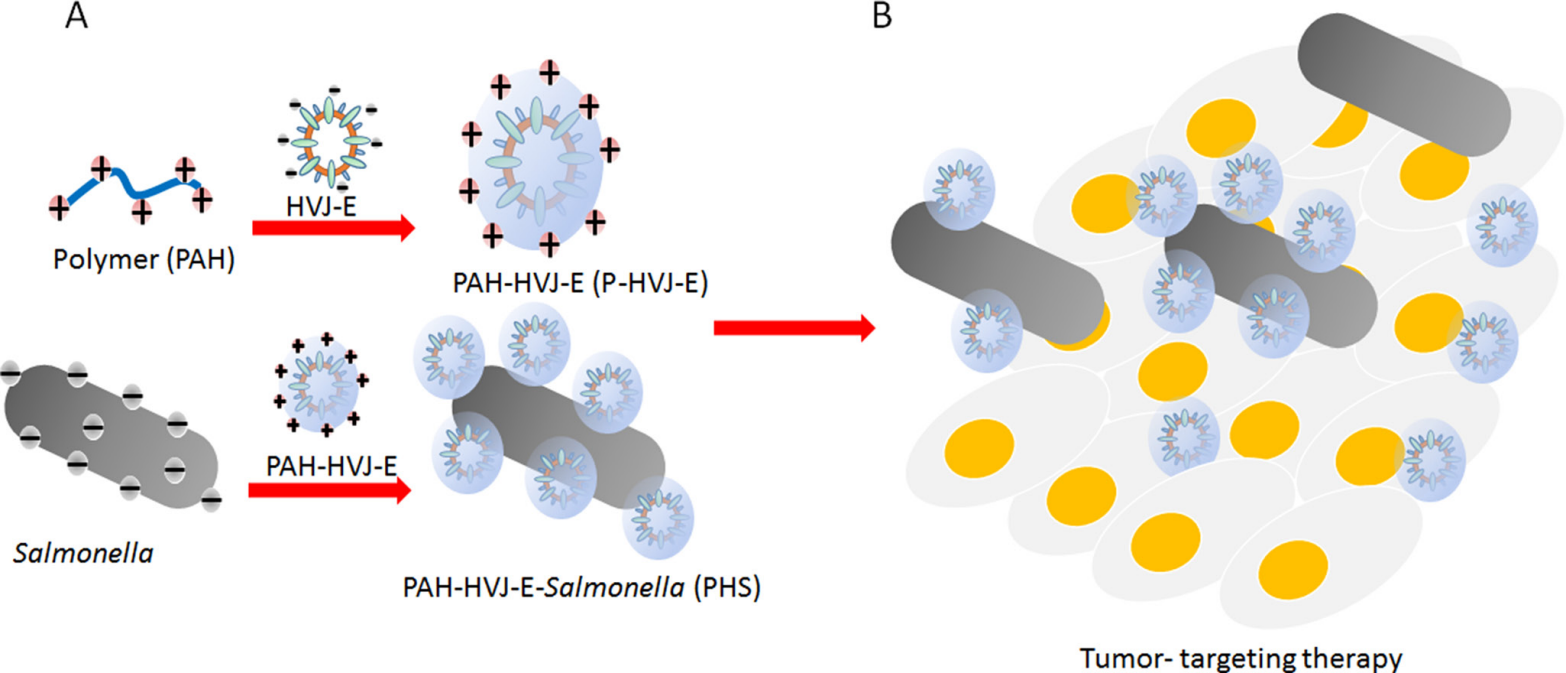
Schematic illustration of the HVJ-E-coated *Salmonella* for improved tumor targeting activity (**A**) Engineering of the HVJ-E-coated *Salmonella***. (B)** Tumor-targeting delivery of the HVJ-E mediated by PAH-HVJ-E-*Salmonella* (PHS).

**Figure 2:**
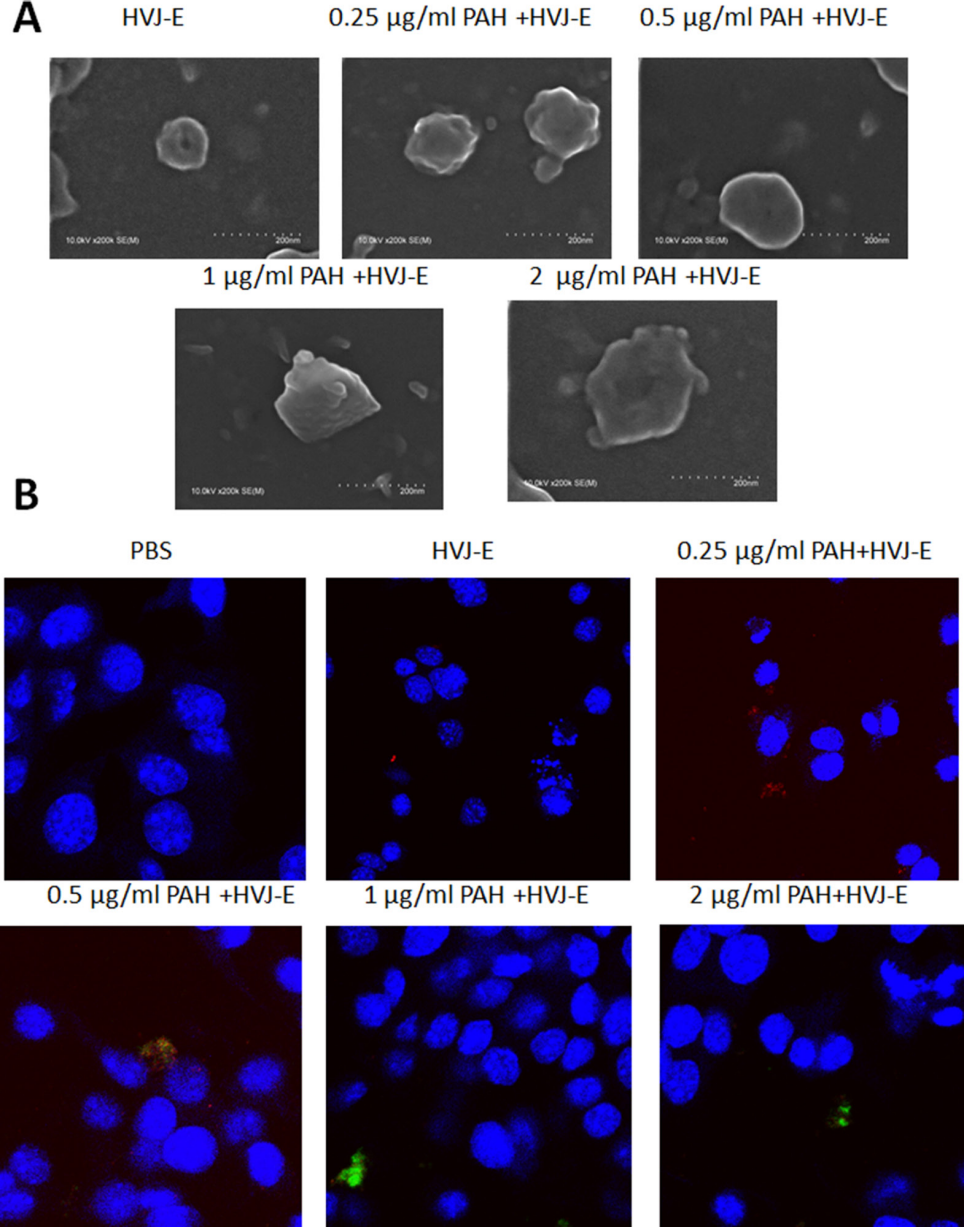
HVJ-E coated with PAH (**A**) Electron microscopy of HVJ-E and of P-HVJ-E (PAH concentrations: 0.25–2 μg/ml). Scale bar = 200 nm (**B**) B16F10 cells treated with 10 MOI of P-HVJ-E were imaged by confocal microscopy. PAH was labeled with fluorescence. HVJ-E was labeled with PKH26 (red). Cell nuclei were counterstained with DAPI.

The hemagglutinin-neuraminidase (HN) protein induced the hemmagglutination after HVJ-E systemic delivery [27–29]. To avoid the hemmagglutination, HVJ-E particles could be packaged into polyelectrolyte shells due to the negative charged of the viral envelope [30]. The HVJ-E-coated *Salmonella* we developed is expected to fulfill the two requirements in systemic HVJ-E delivery and enhance the *Salmonella* antitumor response (Figure 1). Therefore, the positively charged PAH attached to the surfaces of HVJ-E and change the charge of HVJ-E. As shown in Figure 2A, the particle sizes of PAH-modified HVJ-E (P-HVJ-E) with different PAH concentrations were measured by SEM. When compared to the size of unmodified HVJ-E, SEM analysis showed that the size of the P-HVJ-E was significantly increased. The PAH was labeled with FITC (FA-PAH) to demonstrate that PAH masked to the surface of HVJ-E. The HVJ-E was labeled with PKH26 (red). As illustrated in Figure 2B, the HVJ-E was masked with FA-PHA (Figure 2B). The gradual increase in particle size with the concentration of PAH of HVJ-E was evidence that HVJ-E was coated with PAH.

### The characterization of P-HVJ-E

**Figure 3:**
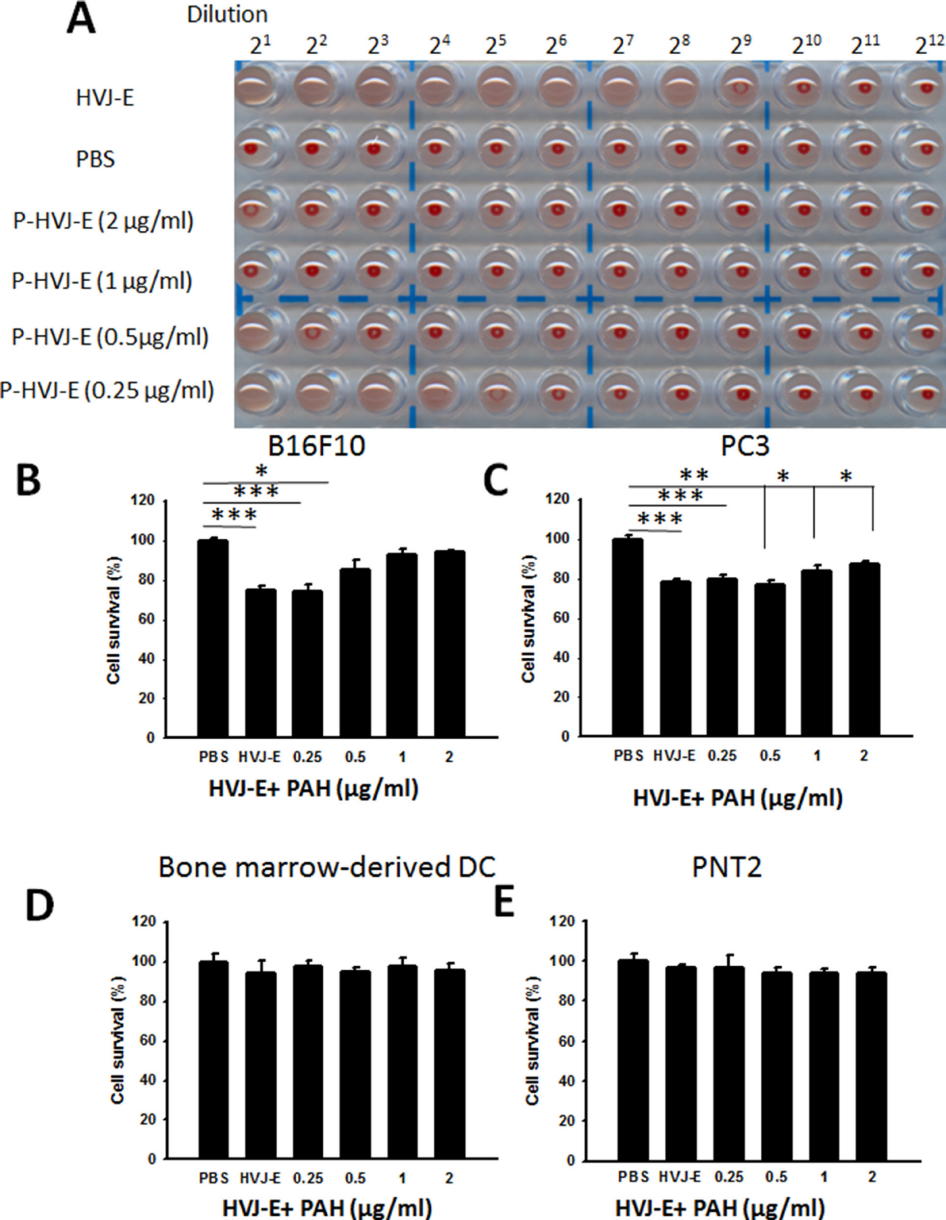
The characterization of P-HVJ-E (**A**) The hemagglutination activity of HVJ-E coated with PAH. Two-fold dilutions of samples of PAH-coating HVJ-E (0- 2 μg/ml) were prepared, mixed with chicken red blood cells, and added to the wells of a 96-well plate. After 30 minutes, the wells were photographed. The cytotoxicity effect of PAH-coating HVJ-E. (**B**) B16F10 (10^5^), (**C**) PC3 (10^5^), (**D**) bone-marrow-derived dendritic cell (10^5^) and (**E**) PNT2 (10^5^) cells were infected with HVJ-E (100 HAU) or HVJ-E coating by various concentrations PAH. The cell viability was then assessed using the WST-1 assay; the data are reported as the means ± SD (*n* = 6). (*P < 0.05; **P < 0.01; ***P < 0.001)

**Figure 4:**
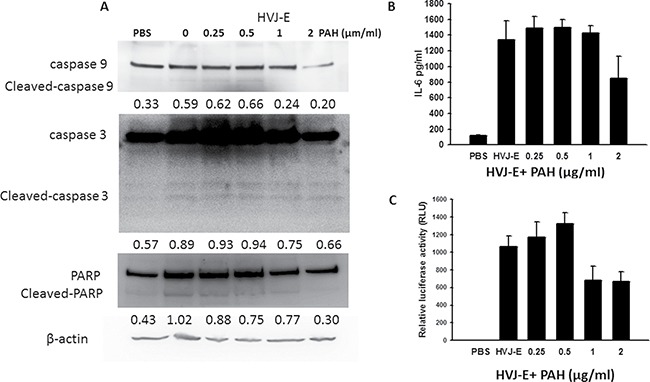
Activity of P-HVJ-E was similar to the activity of HVJ-E (**A**) P-HVJ-E-induced cell death in B16F10 cells mediated by caspase-dependent apoptosis. After exposure to P-HVJ-E (100 HAU) for 24 h, the expression of caspase protein in B16F10 cells was determined by immunoblot analysis. The cropped blots are displayed. The expression of β-actin served as the quantitative control. Inserted values indicated relative proteins expression in comparison with β-actin. (**B**) P-HVJ-E stimulated the expression of IL-6 in bone marrow-derived dendritic cells. After exposure to PAH-coated HVJ-E (100 HAU) for 24 h, the expression of IL-6 in bone marrow-derived dendritic cells was determined by ELISA. (**C**) Luciferase gene expression in B16F10 cells transfected with P-HVJ-E. The P-HVJ-E was incubated with B16F10 cells for 24 h, and the luciferase activity was measured. The data are reported as the means ± SD (*n* = 6).

We used a hemagglutinating assay (HA) to measure the HVJ-E particles and HVJ-E masked with PAH (P-HVJ-E). The hemagglutinating activity inhibits the systemic HVJ-E treatment. When HVJ-E was added to chicken erythrocytes, hemagglutination was induced (Hemagglutinating unit (HAU) 10240/ml) (Figure 3A). However, the hemagglutination activity was significantly decreased in P-HVJ-E group (Figure 3A). Mouse melanoma (B16F10), human prostate cancer cell cells (PC3), mouse bone marrow derived dendritic cell and human normal prostatic epithelial cells (PNT2) were treated with P-HVJ-E and HVJ-E. The proliferation of B16F10 and PC3 was inhibited, and the proliferation of mouse bone marrow-derived dendritic cells and PNT2 cells were not affected, suggesting that P-HVJ-E-mediated cell death is specifically induced in tumor cells (Figure 3B–3E). HVJ-E specifically induced the apoptosis in tumor cells [24]. B16F10 cells treated with P-HVJ-E exhibited apoptosis phenotypes and the expressions of cleaved caspase-9, caspase-3 and PARP were increased (Figure 4A). HVJ-E coated with PAH (0.25 μg/ml~0.5 μg/ml) maintained the antitumor activity. Suzuki et al. demonstrated that HVJ-E stimulated dendritic cells to release interleukin-6 (IL-6) [31] and that IL-6 inhibited the proliferation of regulatory T cell [32]. As shown in Figure 4B, mouse bone marrow-derived dendritic cells secreted IL-6 after the P-HVJ-E treatments. Furthermore, HVJ-E can be used for gene transfer vector *in vitro* and *in vivo* [30]. The luciferase expression was detected to explore the gene transfer activity of P-HVJ-E carrying a luciferase gene (Figure 4C). B16F10 cells treated with P-HVJ-E prepared with 1–2 μg/ml PAH displayed lower luciferase signals than the P-HVJ-E prepared with 0–0.5 μg/ml PAH. However, the expression of luciferase in P-HVJ-E prepared with 0.5 μg/ml PAH was detected. Therefore, HVJ-E prepared with 1–2 μg/ml PAH slightly decreased antitumor ability, and HVJ-E prepared with 0.25 μg/ml PAH still resulted in the hemagglutination. The P-HVJ-E prepared with 0.5 μg/ml PAH was used in the subsequent experiments.

### The characterization of the P-HVJ-E-masked *Salmonella* (PHS)

**Figure 5:**
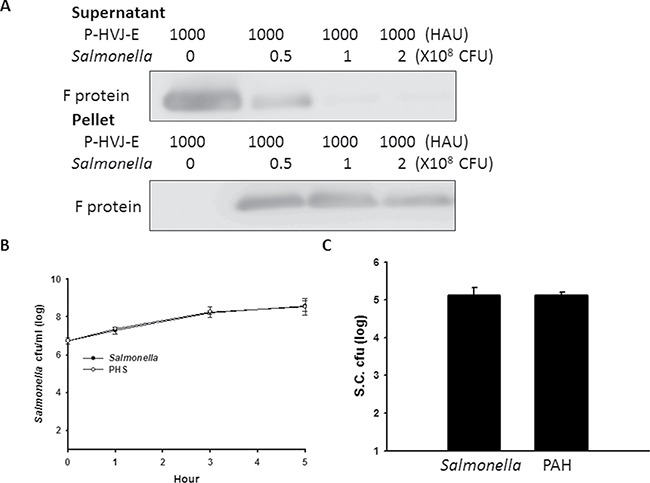
Salmonella coated with P-HVJ-E (PHS) (**A**) P-HVJ-E adhered to the *Salmonella*. PHS cells and supernatant were collected at different concentrations of *Salmonella*. The F proteins of HVJ-E from the solutions were measured using by immunoblot analysis. The cropped blots are displayed. Replication and invasion activity of PHS. (**B**) PHS replication. The number of *Salmonella* and PHS cells was determined 6 h post-incubation. (**C**) A gentamicin protection assay was used to examine these cells 9.5 h later; the data are reported as the means ± SD (*n* = 3).

**Figure 6:**
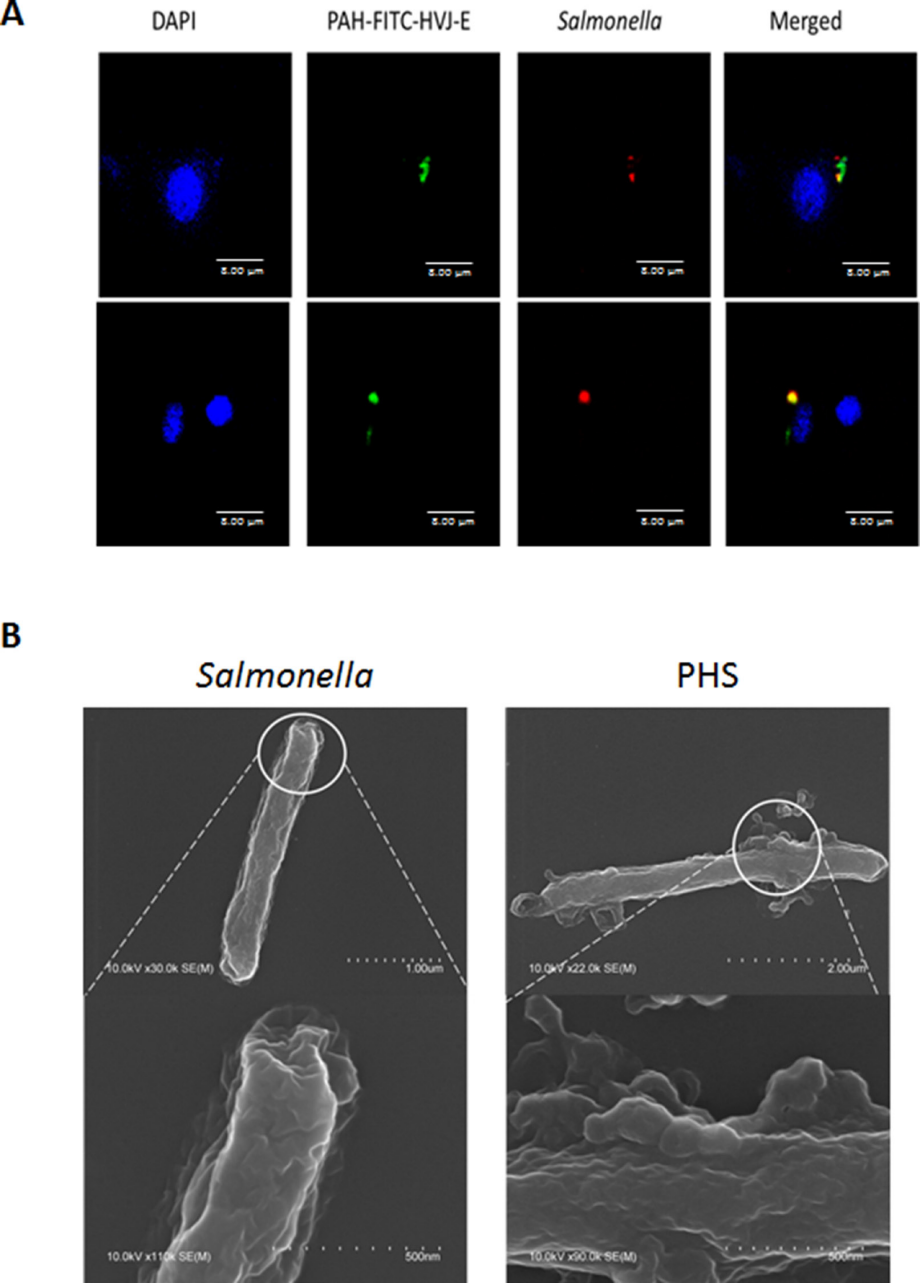
Salmonella coated with P-HVJ-E (**A**) B16F10 cells treated with 10 MOI of PHS were imaged by confocal microscopy. PAH-HVJ-E was labeled with fluorescence. *Salmonella* was labeled with PKH26 (red). Cell nuclei were counterstained with DAPI. Scale bar = 8 μm. (**B**) Electron microscopy of *Salmonella* and of PHS. Upper: scale bar = 1 μm-2 μm. Under: scale bar = 0.5 μm.

**Figure 7:**
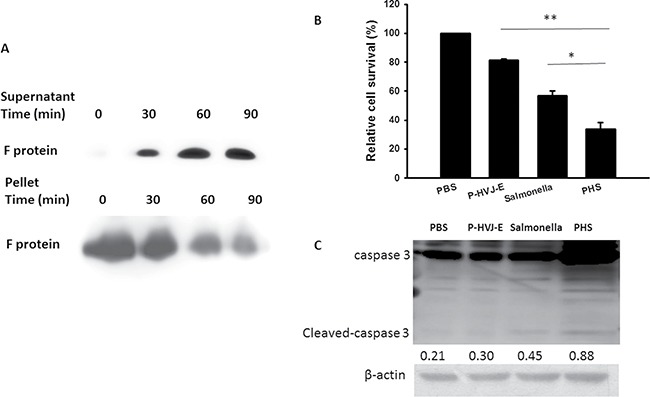
The characterization of PHS (**A**) Kinetics of P-HVJ-E release. PAH cells and supernatants were collected at several time points. The F protein of HVJ-E from the solutions was measured by immunoblot analysis. PHS inhibited the proliferation of B16F10 cells. (**B**) B16F10 cells (10^5^) treated with P-HVJ-E (10 HAU), *Salmonella* (10^6)^ or PHS (10^6^) for 24 h, and the survival rate was then assessed using trypan blue assay. (*P < 0.05; **P < 0.01) (**C**) B16F10 cells (10^5^) treated with P-HVJ-E (10 HAU), *Salmonella* (10^6^) or PHS (10^6^) for 24 h; then, the expression of caspase 3 was assessed using immunoblot assay. The expression of β-actin served as the quantitative control. Inserted values indicated relative proteins expression in comparison with β-actin. The cropped blots are displayed.

The positively charged polyelectrolyte PAH can spontaneously attach on the cell wall of *Salmonella* [25]. Because the F protein of HVJ-E has membrane-fusing potential, HVJ-E used F protein as a cell-fusing or detective agent. To evaluate the number of P-HVJ-E absorbed onto the surface of *Salmonella*, we collected the cell pellet and supernatant at varying numbers in the presence of *Salmonella* followed by the Western blot assay to determine the fusion (F) protein of HVJ-E (Figure 5A). Because one hemagglutination units (HAU) of HVJ-E corresponds to 1 × 10^7^ viral particles [31], one *Salmonella* can absorb 50–100 P-HVJ-E particles. Furthermore, the replication activity of PHS and *Salmonella* were measured to determine whether the P-HVJ-E masking *Salmonella* affected the physiology of *Salmonella*. Meanwhile, the growth curve of *Salmonella* coated with P-HVJ-E (PHS) was not significant difference between *Salmonella* group (Figure 5B). The gentamicin protection/ bacterial invasion assay was used to measure the infection efficiency of PHS cells. As shown in Figure 5C, the invasion efficiency of PHS did not decrease compare with *Salmonella* group (Figure 5C). We used immunofluorescence assay to determine whether the P-HVJ-E particles absorbed onto the *Salmonella* by (Figure 6A). The P-HVJ-E particles were observed on the surface of *Salmonella* by using an electron microscope (Figure 6B). Next, we expected that the P-HVJ-E would be discharged from the surface of *Salmonella* during *Salmonella* replication. To determine P-HVJ-E release *in vitro*, the bacterial pellets and removed supernatants were collected at several time points. The levels of P-HVJ-E decreased during *Salmonella* division (Figure 7A). The antitumor activity of P-HVJ-E, *Salmonella* and PHS against B16F10 cells was examined using a cell viability assay. The PHS significantly inhibited the cell viability of B16F10 (Figure 7B). The expression of cleaved caspase 3 was also significantly observed in the PHS treatment group compared with P-HVJ-E or *Salmonella* groups (Figure 7C). The results suggest that P-HVJ-E could absorb onto *Salmonella* to form PHS and could discharged from *Salmonella* when *Salmonella* underwent replication.

### Tumor-targeting potential of PHS and tumor growth inhibition by PHS

**Figure 8:**
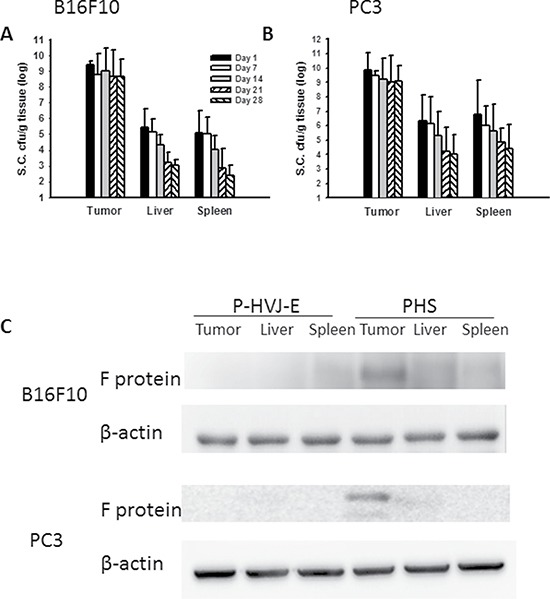
PAH delivered HVJ-E to tumor sites *in vivo* The C57BL/6 and SCID mice that had been inoculated subcutaneously with (**A**) B16F10 (10^6^) and (**B**) PC3 (10^6^) at day 0 were treated i.p. with *Salmonella* (10^6^ CFU/100 μL) at day 8. Mice bearing tumors ranging from 50–100 mm^3^ were injected i.p. with PHS (10^6^ cfu), and the numbers of *Salmonella* in the tumors, livers, and spleens were determined at various time points. The data are reported as the means ± SD (*n* = 4–5); (**C**) B16F10 and PC3 tumor tissues were lysed and the functional protein of HVJ-E was performed on day 1. This experiment was repeated with similar results. The cropped blots are displayed.

**Figure 9:**
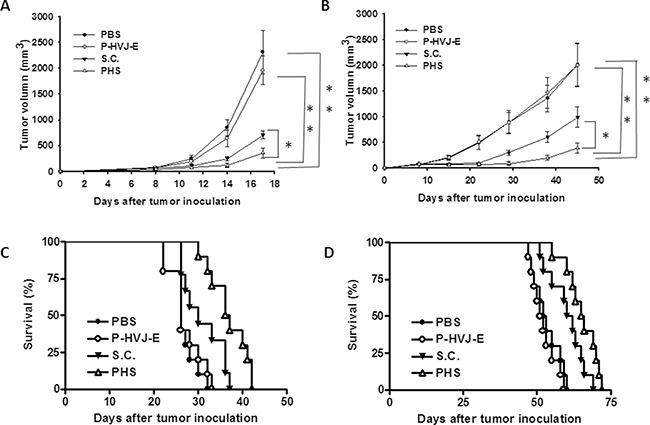
PAH inhibited tumor growth after systemic treatment The B16F10 (**A**) and PC3 (**B**) tumor volumes were measured every 3 days or 7 days after various treatments. This experiment was repeated with similar results. (*Salmonella*; S.C.) (*n* = 10, mean ± SEM. *P < 0.05; **P < 0.01). Kaplan-Meier survival curves of mice bearing (**C**) B16F10 and (**D**) PC3 tumors with different treatments are shown. Data were analyzed by the log-rank test. (*P* < 0.01 for PAH versus PBS and HVJ-E; *P* < 0.05 for S.C. versus PBS and PHS versus S.C.)

The bacteria number of PHS in B16F10 or PC3 tumor-bearing mice was measured after injection with PHS (Figure 8A, 8B). At all the time points examined, the number of *Salmonella* was much higher in tumors than that in the spleens and livers. The number of *Salmonella* in the tumors was 1,000–10,000 times more than that found in the livers or spleens. The number of *Salmonell*a was lesser in the healthy organs compared with that in tumors in both strains of mice at day 28. Meanwhile, to examine whether *Salmonella* delivered P-HVJ-E to the tumor sites, we injected PHS into B16F10 or PC3 tumor-bearing mice and examined the fusion protein of HVJ-E within a tumor, liver and spleen by Western blot assay. The HVJ-E was found in the tumor sites, whereas it was slightly found in the spleen or liver (Figure 8C). The antitumor effect of PHS was evaluated by using B16F10 tumor model. In B16F10 tumor model, the PHS treatment significant inhibited the tumor growth compared with *Salmonella* (*P* = 0.0016), P-HVJ-E (*P* = 0.001) or PBS (*P* = 0.006) treatment. The mean tumor volume of the PHS treatment was lowered by 84%, 82% and 50% in comparison to that in PBS, P-HVJ-E and *Salmonella* treatment, respectively. Furthermore, the delayed tumor growth of PC3-bearing mice treated with PHS was also significantly observed (Figure 9B). The PHS significantly inhibited tumor growth in mice bearing either B16F10 or PC3 tumors (Figure 9A and 9B) and enhanced the survival time (Figure 9C and 9D). *Salmonella* themselves still had strong antitumor activity in immunocompetent and immunodeficient mice and the combination therapy (PHS) showed a stronger antitumor effect.

## DISCUSSION

*Salmonella* target tumors, including those of the bones, prostate, colon, breast, hepatocellular carcinoma, melanoma, pancreas and sarcoma [33–36]. However, the antitumor activity of *Salmonella* varies depending on the tumor. To enhance for this limitation, oncolytic HVJ-E were coating with oppositely charged polymers to adhere to the cell wall of tumor-targeting *Salmonella*.

The attenuated S. *choleraesuis* were used for delivery vectors as DNA vaccine [37]. We have demonstrated tumor-targeting and antitumor activities of attenuated S. *choleraesuis* carrying antiangiogenic genes [23, 38]. S. *choleraesuis* as single-agent therapy can retards tumor progress and prolong survival in mice bearing lung and liver tumors [4, 39]. The combination therapy of S. *choleraesuis* plus low-dose cisplatin in mice bearing subcutaneous tumors showed the additive antitumor effect [4]. The S. *choleraesuis* used in our studies were obtained from the S. *choleraesuis* subsp. choleraesuis serovar Dublin (virulent strain 188) and designated vaccine 51 [40]. We have sequenced the genome of S. *choleraesuis*. Herein, we extend the tumor-targeting function of S. *choleraesuis*.

HVJ-E has a strong antitumor response after intrtumoral injection, but the hemmagglutination limit the HVJ-E administration routes. In the present study, HVJ-E that was encapsulated in a biocompatible polymer increased stability in the tissues and reduced the hemmagglutination [30]. We used PAH to produce shells that covered HVJ-E. Coating HVJ-E with such a shell would not inhibit its antitumor ability and function. The fusion protein of HVJ-E may cover with a high concentration of PAH (1–2 μg/ml) and PAH decreased the antitumor activity of HVJ-E. On the contrary, HVJ-E antigenicity was not covered with a low PAH concentration (0.25 μg/ml) and the HVJ-E antigenicity still resulted in hemmagglutination. PHS successfully delivered HVJ-E and inhibited tumor growth. HVJ-E was found in the tumor tissue after PHS systemic administration (Figure 8C). The applications for HVJ-E in cancer therapy will expand by PHS. Recently, a major challenge for cancer therapy is how to induce immune response in tumor microenvironments. *Salmonella* could not only break tumor immune tolerance, but also accumulate within the tumor region quickly, even leading to tumor cell death. As they have the strong property of activating immunity and killing cancer cells, we believe *Salmonella* are worth studying and applying to anticancer treatments [21]. HVJ-E does not induce interferon (IFN) -γ expression [23]. Previously, we suggested that *Salmonella* stimulated IFN-γ production in T cells and enhanced Th1 immune response through Toll-like receptor 4 signaling [41]. Meanwhile, *Salmonella* reduced the expression of IDO-1 in tumors, which increased infiltrating immune cells such as macrophages, neutrophils and T cells, within the tumor sites [4]. The HVJ-E has the antitumor activity in various types of cancers. In Japan, clinical trials of the safety and antitumor immunity of HVJ-E are under way [24]. The combination therapy of HVJ-E and other immune modulators, such as *Salmonella*, exhibits a more effective activation of antitumor immunity. Orthotopic tumor models is very suitable for evaluating the tumor-targeting activity of *Salmonella* [9]. Some studies and our previous results suggested that *Salmonella* targeted not only in subcutaneous but also in orthotopic tumor models after systemic treatment [42, 43].

To investigate the combinatorial effects using *Salmonella* and oncolytic virus, the *Salmonella* infection in combination with oncolytic virus was investigated for tumors. Tumor growth was suppressed by this combination therapy, suggesting candidates for oncolytic therapy against tumor growth. This study suggested that *Salmonella* combined oncolytic virus, augmenting the antitumor activity.

## MATERIALS AND METHODS

### Bacteria, HVJ-E, cells, reagent and mice

A vaccine strain of S. *Choleraesuis* [S. choleraesuis subsp. choleraesuis (Smith) Weldin serovar Dublin (ATCC 15480)] was obtained from Bioresources Collection and Research Center (Hsinchu, Taiwan). S. *Choleraesuis* carried luciferase gene (S.C./Luc) as previous described [26]. HVJ (Z strain) was propagated in chicken eggs. The virus was inactivated by UV irradiation (99 mJ/cm^2^) immediately prior to each experiment. Viral replication was completely eliminated by UV irradiation. Human PC3 (prostate cancer), PNT2 (prostatic epithelial cells) and B16F10 (melanoma) cells were cultured in Dulbecco's modified Eagle's medium containing 10% fetal bovine serum, 1% glutamine, and 50 μg/ml gentamicin at 37°C in 5% CO2. Mouse bone marrow-derived dendritic cells were collected from mouse tibias and femurs and treated with granulocyte-macrophage colony-stimulating factor (GM-CSF) (Sigma-Aldrich, St Louis, MO, USA). Bone marrow-derived dendritic cells were ready for experimental use. Polyallylamine hydrochloride (PAH, MW=15, 000), fluorescence-PAH, and PKH26 were purchased from Sigma (Sigma-Aldrich). The HVJ-E was stained with PKH26 according to the manufacturer's instructions and examined under a fluorescence microscope. The levels of cytokines, interleukin-6 (IL-6) in the supernatant of cells after HVJ-E or P-HVJ-E administration was determined by enzyme-linked immunosorbent assay (ELISA) (R & D, Minneapolis, MN, USA). In the subcutaneous tumor models, mice were injected with B16F10 (10^6^) or PC3 (10^6^) into the left flank. For the evaluation of the tumor-targeting potential of *Salmonella* in tumors, the bacteria present in tumors, blood, liver, and spleen were determined by plating serial dilutions of the homogenates onto LB agar plates, incubating overnight at 37°C, and counting bacterial colonies. C57BL/6 mice or severe combined immunodeficiency (SCID) mice were purchased from the National Laboratory Animal Center of Taiwan. The animals were maintained in a pathogen-free animal care facility in isothermal conditions with regular photoperiods. The experimental protocol adhered to the rules of the Animal Protection Act of Taiwan and was approved by the Laboratory Animal Care and Use Committee of the China Medical University (permit number: 101–20-N). Groups of 10 C57BL/6 mice or SCID mice were inoculated with B16F10 or PC3 (10^6^) cells. After 7–9 days, when the tumors were approximately 50–100 mm^3^, *Salmonella* (10^6^ cfu), P-HVJ-E (100 HAU), PHS (10^6^ cfu) or PBS was injected intraperitoneally (i.p.) in mice bearing B16F10 or PC3 tumor cells. To analyze tumor volumes, the tumors were measured every 3 days or 1 week in two perpendicular axes using a tissue caliper, and the tumor volumes were calculated as (length of tumor) × (width of tumor)^2^ × 0.45. All of the mice were monitored for tumor growth and survival. For the evaluation of the tumor targeting potential of HVJ-E in tumors, the viral particles present in tumors, liver or spleen were determined by immunoblotting assay after 1 day PHS (10 ^6^ cfu) treatment.

### Hemagglutination assay

The hemagglutination assay was performed in a 96-well round-bottom plate using 50 μl/well of a 0.5% suspension of chicken, red blood cells and 50 μl/well of a HVJ-E solution serially diluted with PBS.

### Gentamicin protection assay, trypan blue assay and luciferase assay

Cells were infected with *Salmonella* or PHS after 2 h incubation; the medium was changed to medium containing 50 μg/ml gentamicin for 1 h. This were followed by two washes with warm PBS and lysis of cells using 0.5% (v/v) Triton-X-100/PBS for 20 min on ice. Serial dilutions were plated on LB agar and incubated overnight at 37°C; then, the bacterial colonies were counted. Cells (10^5^/well) were infected with 2 × 10^6^ cfu of *Salmonella*, PHS or mock-infected with antibiotic-free culture medium for 2 h. The medium were removed, washed, and replenished with fresh medium supplemented with 2% FBS and 50 μg/ml gentamicin. Cell survival was assessed using the trypan blue exclusion assay [44]. Subsequently 10^6^ cfu of S.C./Luc or PHS/Luc were added to these cells which were cultured in 1 ml of antibiotic-free medium and incubated for 8 h. All the cells were washed, replenished with gentamicin (50 μg/ml)-containing complete medium, and further cultured for 16 h. Cells were ysed to prepare cell extracts for determining luciferase activity by a luciferase assay kit (Tropix, Bedford, MA).

### Scanning electron microscope (SEM)

HVJ-E, P-HVJ-E and PHS were fixed with 2.5% glutaraldehyde in phosphate buffer and then fixed in 1% OsO_4_ solution for 1 h. The samples were dehydrated in a graded ethanol series and embedded in Quetol 812 epoxy resin (Nissin EM, Tokyo, Japan). The samples were examined under a Hitachi electron microscope (Hitachi, Tokyo, Japan).

### Western blot analysis

The protein content in each sample was determined by the bicinchoninic acid (BCA) protein assay (Pierce Biotechnology, Rockford, IL). Then, 60–80 μg of protein with 4 × SDS sample dye added was denatured by heating at 10 min at 95°C. Proteins were fractionated on SDS-PAGE, transferred onto Hybond enhanced chemiluminescence nitrocellulose membranes (Amersham, Little Chalfont, UK) and detected with antibodies against caspase 3 (Cell Signaling, Danvers, MA), caspase 9 (Cell Signaling), Poly (ADP-Ribose) polymerase (PARP) (Cell Signaling), anti-F (fusion protein of HVJ) (Hokkaido System Science Co., Ltd, Hokkaido, Japan) and β-actin (Sigma Aldrich). Rabbit anti-mouse IgG-peroxidase antibody (Sigma Aldrich) and donkey anti-rabbit IgG-peroxidase antibody (Sigma Aldrich) were used as the secondary antibody, and protein-antibody complexes were visualized by an enhanced chemiluminescence system (GE Healthcare, UK). The signals were quantified with ImageJ software (rsbweb.nih.gov/ij).

### Statistical analysis

The one-way analysis of variance (one-way ANOVA) was used to determine differences between groups for comparison to the control group. The survival analysis was performed using the Kaplan-Meier survival curve and log-rank test. A *P* value less than 0.05 was considered to be statistically significant. (*P < 0.05; **P < 0.01; ***P < 0.001).
